# Clinical Implication of Patchy Pattern Corneal Staining in Dry Eye Disease

**DOI:** 10.3390/diagnostics11020232

**Published:** 2021-02-03

**Authors:** Seitaro Komai, Norihiko Yokoi, Hiroaki Kato, Aoi Komuro, Yukiko Sonomura, Shigeru Kinoshita, Chie Sotozono

**Affiliations:** 1 Department of Ophthalmology, Kyoto Prefectural University of Medicine, Kyoto 602-8566, Japan; s-koma@koto.kpu-m.ac.jp (S.K.); hiro-kat@koto.kpu-m.ac.jp (H.K.); akomuro@koto.kpu-m.ac.jp (A.K.); yukky@ymail.plala.or.jp (Y.S.); csotozon@koto.kpu-m.ac.jp (C.S.); 2 Department of Frontier Medical Science and Technology for Ophthalmology, Kyoto Prefectural University of Medicine, Kyoto 602-8566, Japan; shigeruk@koto.kpu-m.ac.jp

**Keywords:** dry eye, fluorescein corneal staining, superficial punctate keratopathy, Sjögren’s syndrome

## Abstract

Corneal fluorescein staining in a form that is commonly called a “patchy pattern (PP)” is sometimes seen with or without superficial punctate keratopathy (SPK) in dry-eye diseases (DEDs). Here, we investigated the differences in the clinical features of DED patients with and without PP corneal staining (PPCS). This study involved 35 DEDs with PPCS (PPCS group) and 30 DEDs with SPK and without PPCS (non-PPCS group). The tear meniscus radius (TMR, mm), spread grade (SG) of the tear-film lipid layer (i.e., SG 1–5, 1 being best), noninvasive breakup time (NIBUT, seconds), fluorescein breakup time (FBUT, seconds), corneal epithelial damage (CED, 15 points maximum), conjunctival epithelial damage (CjED, six points maximum), the Schirmer’s 1 test (ST1, mm), and the prevalence of Sjögren’s syndrome (SS) were examined, and then compared between the two groups. Our findings revealed that between the groups (PPCS vs. non-PPCS), there was a statistically significant difference (*p* < 0.05) in CjED (3.1 ± 1.9 vs. 1.3 ± 1.6), ST1 (5.6 ± 7.4 vs. 14.8 ± 11.4), and the prevalence of SS (60.0% vs. 16.7%). Our findings suggest that DEDs and dry-eye patients with PPCS may indicate not only SS itself, but also the ophthalmological characteristics compatible with SS.

## 1. Introduction

Superficial punctate keratopathy (SPK) is a corneal epithelial abnormality clinically characterized by the punctate staining of the corneal surface when observed via fluorescein-assisted slit-lamp biomicroscopy. SPK is observed in a variety of ocular surface diseases, such as dry eye diseases (DEDs) [1], the eyes of contact lens wearers [2,3], drug-induced epithelial keratopathy [4], and ocular trauma, as well as in normal eyes [5]. As for the pathology of SPK, some previous reports have suggested that SPK may be the pooling of dye within the space resultant from desquamated epithelial cells [1,6,7], or the uptake of dye into superficial epithelial cells [1,6,8,9,10].

In DEDs, the distribution and density of SPK has been used as an index of severity of the disease [11,12,13,14] in which the horizontal and/or interpalpebral distribution of SPK is known to be characteristic to DEDs in general, and the lower distribution of SPK may indicate the association with aqueous tear-deficient dry eye diseases (ATDDEs) [13].

**Figure 1:**
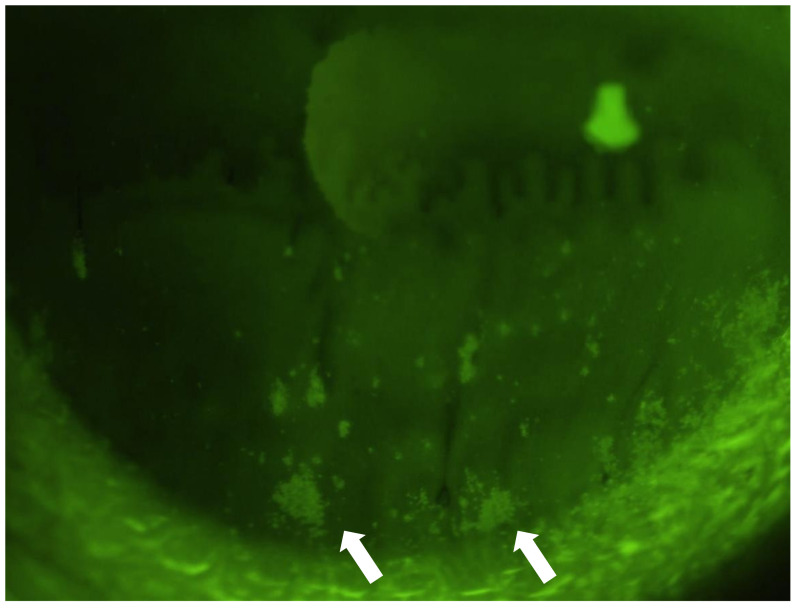
Slit-lamp photograph of fluorescein “patchy pattern” corneal staining (PPCS). PPCS can be seen on the lower cornea (white arrows) as a mottled-pattern staining, quite unlike ordinary superficial punctate keratopathy (SPK).

Corneal fluorescein staining in a form that is commonly called a “patchy pattern (PP)” (Figure 1) is sometimes observed with or without SPK in patients afflicted with DED. Similar to PP corneal staining (PPCS), corneal mucus plaques (CMPs) are sometimes seen in patients with relatively severe ATDDEs [15,16]. CMPs are aggregates of various sizes and shapes that attach to the corneal surface, and they are mainly composed of mucus, epithelial cells, and proteins [17]. For the conjunctiva as well, the periodic acid-schiff-positive mucous aggregation has been reported in Sjögren’s syndrome (SS) patients [18]. Although it is speculated that the pathology of PPCS corresponds to the attachment of a dried mucous component on the corneal surface with or without damaged surface epithelium, the clinical implication of PPCS has yet to be elucidated.

Therefore, the purpose of this present study was to investigate the differences in the clinical features in DED patients with and without PPCS.

## 2. Materials and Methods

### 2.1. Study Participants

This retrospective study was approved by the Institutional Review Board of the Kyoto Prefectural University of Medicine (KPUM), Kyoto, Japan, (project identification code RMBR-C-625-1 and 25. 11. 2009 of approval) and was conducted in accordance with the tenets set forth in the Declaration of Helsinki. Prior written informed consent was obtained from all subjects. To protect each subject’s personal safety, human rights, and privacy, all personal information was kept in strict confidence, great care was taken in the handling of the collected data, and all personally identifiable information was excluded from the data to be published. Moreover, the data were never used for any other purpose than in this study.

This study involved subjects who were enrolled from the KPUM Dry Eye Clinic between May 2013 and May 2015, with the inclusion criteria being patients diagnosed with DED and who underwent fluorescein corneal staining. Briefly, eyes with more severe subjective symptoms were enrolled, and in the cases in which the same level of severity was observed in both eyes, and the left eye data were used. The exclusion criteria were as follows: (1) cases with eyelid diseases such as ptosis, blepharospasm, lagophthalmos, entropion, and ectropion; (2) cases with a history of eye surgery other than cataract surgery; (3) cases diagnosed with meibomian gland dysfunction (MGD) based on the Japanese diagnostic criteria for MGD; (4) cases with severe conjunctivitis, upper limbal keratoconjunctivitis, lid wiper epitheliopathy, or filamentary keratitis and whose symptoms could be explained by these conditions alone; and (5) cases deemed inappropriate for inclusion in this study, such as those with ocular surface diseases other than dry eye, or those in which less than 3 months had passed since undergoing cataract surgery. These subjects were excluded from the analysis with the consent of the evaluators (N.Y., H.K., A.K., Y.S., S.K.). Since the purpose of this study was to determine the relationship between PPCS and the characteristics of patients with DED, diseases that excessively interfere with the eyelid and ocular surface were excluded. Although the subjects in this study did not receive systemic treatment for DED, topical treatments including preservative-free artificial tears (6 times daily), 0.1% hyaluronic acid (4–6 times daily), 3% diquafosol sodium eye drops (4–6 times daily), and/or 2% rebamipide eye drops (4 times daily), and/or 0.1% fluorometholone (twice daily) were administrated according to the fluorescein breakup pattern [19] and the severity of symptoms. In order to prevent any possible effect of eye drop use, all subjects were instructed to discontinue use for at least 1 h prior to examination.

### 2.2. Fluorescein Corneal Staining Findings

**Figure 2:**
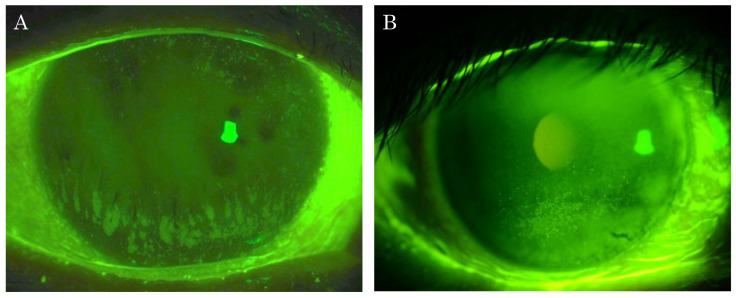
Representative photographs of the fluorescein patchy pattern corneal staining (PPCS) and superficial punctate keratopathy (SPK) without PPCS. PPCS can be seen at the lower part of the cornea (**A**) and SPK without PPCS can also be seen at the lower part of the cornea (**B**).

According to the objective fluorescein corneal staining findings, then enrolled subjects were classified into either the group with PPCS (PPCS group) or the group with SPK and without PPCS (non-PPCS group). PPCS was defined as the fluorescein staining that was densely distributed in a mottled pattern unlike ordinary SPK. Subjects were classified as a PPCS group when at least one PPCS was observed, even if the appearance of other staining corresponded to ordinary SPK (Figure 2A). SPK without PPCS was diagnosed only when all staining corresponded to ordinary SPK (Figure 2B). In all cases, the density or range of SPK was not considered in the classification. Under the agreement of 3 ophthalmologists (S.K., N.Y., H.K.), all cases were evaluated from the photographs obtained after fluorescein staining using a slit-lamp microscope.

### 2.3. Ocular Surface Evaluations

Ocular surface evaluations were performed in the order from noninvasive to invasive examinations so as not to affect subsequent examinations. Moreover, the association of SS was diagnosed based on Fox’s criteria [20].

#### 2.3.1. Noninvasive Assessment of Tears

First, noninvasive tear film evaluation, including measurements of the tear meniscus radius (TMR, mm), spread grade (SG) of the tear-film lipid layer (TFLL) [21], and the noninvasive breakup time (NIBUT, in seconds) [22,23] of the ocular surface tear film, was performed. The TMR of the central lower tear meniscus was measured using a video-meniscometer [22,24,25,26]. The TMR is a parameter reflecting the total aqueous tear volume over the ocular surface. An illuminated target with horizontal stripes was projected onto the tear meniscus at the central lower lid margin. A digital video recorder was used to record the specular image of the target, and the radius of the tear meniscus curvature (mm) was calculated by image analysis software based on a concave mirror formula.

SG and NIBUT were evaluated via the use of a DR-1^®^ video-interferometer (Kowa Company, Ltd., Tokyo, Japan) after several natural blinks. On this examination, the central TFLL of the cornea can be observed within the central 6.8 mm (vertical) × 8.8 mm (horizontal) area of the cornea. The SG of the TFLL is graded from 1 to 5 depending on the degree of upward spread of the TFLL: (1) G1: quick and complete (the spreading TFLL quickly reaching the upper lid margin); (2) G2: slow and complete; (3) G3: slow and partial (the spreading TFLL not reaching the upper lid margin but reaching to >1/2 the height of the image); (4) G4: slow and partial (the spreading TFLL reaching ≤1/2 the height of the image); and (5) G5: no spreading of TFLL. After the evaluation of SG was performed, NIBUT was evaluated. NIBUT was measured as the time (in seconds) until the first full-thickness breakup of the tear film (noninvasive breakup of tear film) was observed by keeping the eye open for up to 10 s. When the breakup of tear film was not observed until 10 s, NIBUT was recorded as 10 s. NIBUT was measured only once in order to prevent reflex tearing that may interfere with the following examinations.

#### 2.3.2. Measurement of Fluorescein Breakup Time (FBUT) and Scoring of Ocular Surface Epithelial Damage

Measurement of fluorescein breakup time (FBUT) and scoring of ocular surface staining were performed using a slit-lamp microscope with a cobalt blue filter and blue-free filter [27] after the staining of tears with sodium fluorescein. For the fluorescein staining, a fluorescein test strip (Showa Yakuhin Kako Co., Tokyo, Japan) was softly touched to the central lower lid margin to prevent increasing the subject’s tear volume. After several natural blinks, the time (in seconds) until the first appearance of fluorescein breakup of the precorneal tear film by keeping the eye open was recorded. FBUT was measured 3 times, and then averaged.

Corneal epithelial damage (CED) was evaluated in accordance with the National Eye Institute (NEI) scoring system [12]. The cornea was divided into 5 areas, and the corneal staining at each area was scored from 0 to 3 depending on the severity of the staining. In this scoring, at each area, PPCS was regarded as densely distributed focal SPK, as PPCS is also thought to be a kind of epithelial damage. The total score of the 5 areas was defined as CED. Conjunctival epithelial damage (CjED) was evaluated based on the van Bijsterveld scoring system [14]. The nasal and temporal bulbar conjunctiva were scored from 0 to 3 depending on the severity of the staining at each area, and the total score was calculated. CED and CjED were assessed on a range of 0–15 and 0–6 points, respectively.

#### 2.3.3. Additional Assessments of the Ocular Surface

Finally, the Schirmer 1 test (ST1, mm) was performed 10 min after the evaluation of the ocular surface staining without topical anesthesia. Tear secretion for 5 min under natural blinking was measured and determined by the length of the wetted filter paper (Whatman No. 41; Showa Yakuhin Kako, Tokyo, Japan). The length of the wetted portion of the paper was recorded in millimeters.

### 2.4. Statistical Analysis

For statistical analyses, the unpaired t-tests were used for TMR and ST1. The Wilcoxon rank sum test was used for SG, NIBUT, FBUT, and the ocular surface epithelial staining scores. The chi-square test was used for analysis of the prevalence of SS. All statistical analyses were performed using JMP software version 14.0.0 (SAS Institute Inc. Cary, NC, USA). A *p*-value of <0.05 was considered statistically significant.

## 3. Results

This study involved 65 eyes of 65 DED patients (61 females (F) and four males (M); 35 right (R) eyes and 30 left (L) eyes; mean age: 65.2 ± 9.7 (43–83) years (mean ± SD)). Via the corneal fluorescein staining-assisted slit-lamp biomicroscopy examination of the 65 subjects, 35 eyes were classified as DED with PPCS (PPCS group) and 30 eyes as DED with SPK and without PPCS (non-PPCS group).

There was no statistically significant difference for each group (PPCS group; non-PPCS group) in terms of sex (*p =* 0.38) ((M, F; number of cases): (3; 32) and (1; 29)), laterality (*p* = 0.94) ((R, L; number of cases): (19; 16) and (16; 14)), and age (*p =* 0.21) (66.7 ± 8.0; 63.5 ± 11.3; years).

**Figure 3:**
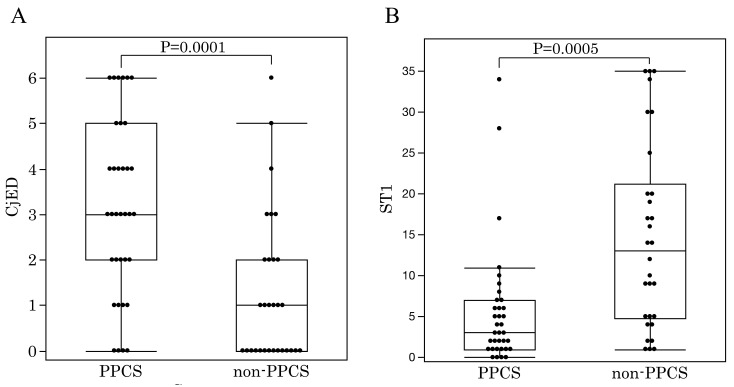
(**A**) Conjunctival epithelial damage (CjED) and (**B**) Schirmer I test (ST1) results in the patchy pattern corneal staining (PPCS) group and non-PPCS group. There was a statistically significant difference in both results between the two groups.

The respective results (PPCS group; non-PPCS group) for TMR, SG of the TFLL, NIBUT, FBUT, CED, CjED, ST1, and the prevalence of SS were (TMR: 0.18 ± 0.08; 0.22 ± 0.09), (SG: 16, 4, 6, 6, 3; 20, 6, 0, 1, 3, the number of cases, respectively, for grade 1, 2, 3, 4 and 5), (NIBUT: 3.0 ± 2.2; 4.4 ± 3.4), (FBUT: 1.8 ± 1.3; 2.9 ± 2.8), (CED: 5.3 ± 3.9; 4.3 ± 4.5), (CjED: 3.1 ± 1.9; 1.3 ± 1.6), (ST1: 5.6 ± 7.4; 14.7 ± 11.4), and (prevalence of SS: 60.0%; 16.7%) (Table 1). In the comparison of the results of each examination in the PPCS and non-PPCS groups, a statistically significant difference was found in CjED (*p* = 0.0001), ST1 values (*p* = 0.005), and the prevalence of SS (*p* = 0.0004), yet no statistically significant difference was found in TMR, SG, NIBUT, FBUT and CED (Figure 3).

## 4. Discussion

The findings in this study revealed statistically significant differences in CjED, ST1, and the prevalence of SS between the PPCS group (DEDs with PPCS) and the non-PPCS group (DEDs with SPK and without PPCS); however, no significant differences in TMR, SG, NIBUT, FBUT and CED between the two groups was observed, possibly due to the following reasons.

First, between the PPCS group and non-PPCS group, there was no significant difference in TMR and SG, thus suggesting that there is no significant difference in tear volume at the ocular surface, as both indicators are reported to reflect tear volume at the ocular surface [22,28]. However, considering that the averaged TMR in both groups was lower than that in normal eyes [25], we theorized that both groups had a similar degree of reduced tear volume. In addition, the finding that there was no significant difference in both NIBUT and FBUT between the two groups can be understood simply from the former result that there was no significant difference in TMR between the two groups. Since the thickness of the aqueous layer of the precorneal tear film is reportedly proportional to TMR [29], there was, therefore, equivalent TMR results in the equivalent thickness of aqueous tear film leading to equivalent tear film stability. On the other hand, ST1 was significantly lower in the PPCS group than in the non-PPCS group. This finding is compatible with the greater prevalence of SS in the PPCS group, since ST1, which reflects reflex tear secretion [30], tends to be lower in DEDs associated with SS because of the functional and organic abnormalities in the lacrimal gland in SS [31]. Accordingly, from the aspect of the quantity and quality of the tear film, SS and SS-associated lacrimal gland dysfunction may possibly be involved in the manifestation of PPCS.

Second, the comparison of the ocular surface epithelial damage between the PPCS group and non-PPCS group showed that CjED was significantly more severe in the PPCS group, despite the fact that no significant difference in CED was found between the two groups. In our previous study that focused on the factors that determine corneal and conjunctival staining scores in DEDs, we found that a greater SG and a shorter FBUT were related to both corneal and conjunctival staining scores, while a lower ST1 and a greater upper eyelid closing-phase maximum velocity were related to only conjunctival staining scores [32]. Therefore, the result that there was no significant difference in CED between the PPCS and non-PPCS group can be explained by the finding that there was no significant difference in SG and FBUT in this study. On the other hand, the greater CjED in the PPCS group can be explained by our previous report that a greater CjED is associated with a lower ST1 value, as stated above, because the ST1 value was less in the PPCS group than in the non-PPCS group. In addition, the greater association of SS in PPCS then also becomes an explanation for the greater CjED, as it has been reported that squamous metaplasia of the conjunctival epithelium is more likely to be associated with SS [33,34]. Namely, the expected dysfunction of membrane-associated mucin of the conjunctival surface epithelium, which may result in the increase in blink-related friction between the eyelid and the bulbar conjunctiva, could also have contributed to the CjED in PPCS group. In this present study, blink-related parameters were not evaluated. However, and as was previously reported [32], we theorize that blink-related parameters may be abnormal in the PPCS group. As stated above, from the point of ocular surface epithelial damage, SS was also thought to be associated with the manifestation of PPCS. Therefore, together with the results of the examination of the tears and tear film, it strongly suggests that PPCS may be associated with SS.

In order to discuss the mechanism of PPCS development in DEDs associated with SS, the pathology of the SS-related DEDs should be considered. The rose Bengal staining of the conjunctival epithelium is a characteristic finding in SS-related DEDs, and rose Bengal dye is known to stain the areas of disruption of mucin MUC16 [35,36], a type of membrane-associated mucin that is expressed at the top of the microvilli of corneal and conjunctival epithelium [37]. Normally, MUC16 helps maintain the barrier function [35,38] and wettability [36] of the corneal and conjunctival epithelium. However, in SS-related DEDs, MUC16 is cleaved from the cell surface and released into the tear fluid (shedding) by pro-inflammatory mediators such as TNF-α and MMP-9, which are increased in the tear fluid in SS [39,40,41]. In other words, the increased shedding of MUC16 in SS-related DEDs may result in impaired barrier function and the decreased wettability of the superficial epithelium. Therefore, although rose Bengal staining was not performed in this present study, based on the significant involvement of SS in the PPCS group, we speculate that the shedding of MUC16 from the corneal and conjunctival surface epithelium was increased, thus resulting in impaired barrier function and decreased wettability, especially in the cases with SS in the PPCS group.

The pathogenesis of CMP, which is similar to PPCS in its manifestation, should also be considered when investigating the mechanism of PPCS development. CMPs are also manifested as abnormal staining with fluorescein at the corneal surface, and vary in size and shape [17,42]. Furthermore, CMP is reportedly more common in ATDDE associated with SS [18,42], which is consistent with our finding that SS is associated with the PPCS group, thus possibly suggesting that PPCS may be a kind of CMP. Since CMP is an aggregate composed of mucin, epithelial cells, protein, and lipids attached to the cornea [17], PPCS is also considered to be aggregates containing mucin. Mucin has a characteristic of forming complexes by trapping various components within its network structure, and it is speculated that those aggregates are increased by the increase in mucin in tears. Therefore, consideration of the turnover of mucin may provide some insight into the possible mechanism of PPCS. The concentration of MUC16 is reportedly increased in the tear fluid of DEDs associated with SS [43]. This increase in MUC16 is generally thought to be due to two mechanisms, ocular surface inflammation and delayed tear clearance associated with SS-related ATDDEs. In SS-related ATDDEs, inflammatory mediators such as MMP-9 and TNF-α are reportedly increased in tears [39,40,41], which results in an acceleration of the shedding of MUC16 which leads to the increase in MUC16 concentration in tears. Also, delayed tear clearance associated with ATDDEs may not only lead to the accumulation of MUC16 in tears, but also the accumulation of inflammatory mediators in tears which may also facilitate the shedding of MUC16. Accordingly, we theorize that the increase in MUC16 in tears is one of the mechanisms that contributed to the corneal manifestation of PPCS in the PPSC group via the formation of mucin aggregates.

On the other hand, the contribution of MUC5AC, the secretory mucin, to the manifestation of PPCS remains unclear, as MUC5AC is reportedly reduced in the tears of SS DEDs compared to tears in normal eyes [44,45,46]. However, and to the best of our knowledge, there have been no reports on the difference of MUC5AC concentration between SS and non-SS DEDs. In comparison with non-SS DEDs, it is thought that in SS DEDs, MUC5AC may also be accumulated in tears due to delayed tear clearance, although MUC5AC may also be decreased due to the reduced number of goblet cells associated with SS [44,46].

Electrostatic attraction between the corneal surface and mucin aggregates may also contribute to the manifestation of PPCS. That is because due to the shedding of MUC16, which is a negatively charged macromolecule [37], the corneal surface becomes less negatively charged, and therefore, repulsive forces between the mucin in tears and the corneal surface may be attenuated, thus resulting in the possible facilitation of the attachment of mucin aggregates to the corneal surface.

As another possible mechanism, blink-related increased friction associated with aqueous tear deficiency may be involved in the manifestation of PPCS, similar to the mechanism for filamentary keratitis, in which mucins MUC16 and MUC5AC surround and adhere to the epithelial cells as a core of the filament [47]. Tears work as a lubricant during blinking, and therefore, in ATDDEs associated with SS, especially on the corneal surface in which wettability is reduced due to the shedding of MUC16, blink-related friction must be increased and the adherence of a mucin component is likely to result in PPCS [48].

**Figure 4:**
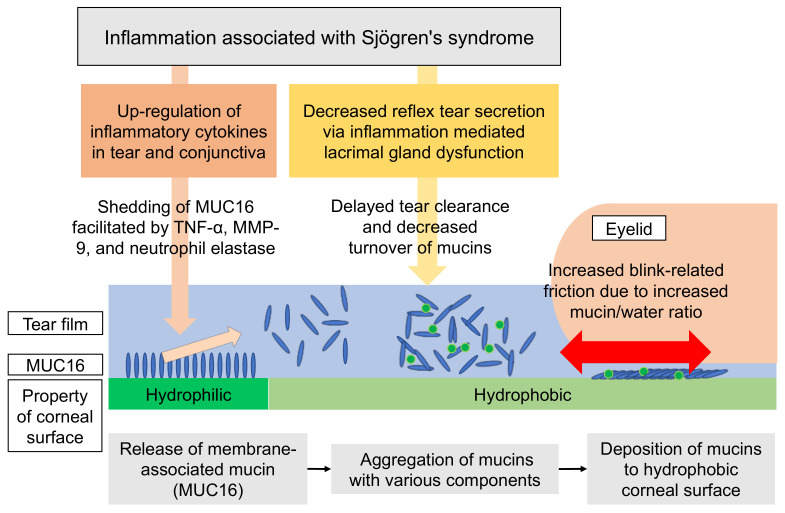
Proposed mechanism in PPCS. Inflammation associated with Sjögren’s syndrome (SS) results in the production of inflammatory cytokines in tears and the conjunctiva. As a result, those cytokines, including TNF-α, MMP-9, and neutrophil elastase, may cause the shedding of mucin MUC16 from the ocular surface epithelium, thus leading to the accumulation of the mucin components in tears. This accumulation of mucins in tears is accelerated by delayed tear clearance associated with aqueous tear deficiency (ATD) commonly seen in SS. On the other hand, the physical property of the corneal surface is likely to change from hydrophilic to hydrophobic due to the decrease in MUC16 via shedding, which may lead to the deposition of mucins to the corneal surface. This mechanism may also be enhanced by the increased blink-related friction associated with ATD.

To summarize, the possible mechanisms responsible for the manifestation of PPCS are the increase in MUC16 concentration in tears via the shedding of MUC16 accelerated by ocular surface inflammation associated with SS and the accumulation of mucins due to delayed tear clearance, the reduction in repulsive forces between the corneal surface due to the shedding of MUC16 and the increased mucins in tears, and the increased friction due to aqueous tear deficiency in SS (Figure 4). Although ocular surface inflammation was not evaluated in this study, an inflammation similar to SS may be responsible for the manifestation of PPCS in DEDs unassociated with SS. Through the findings obtained from this present study, it may be suggested that PPCS is a sign of ocular surface inflammation and that anti-inflammatory treatment is an effective therapy for DEDs with PPCS. Therefore, PPCS may become the key for determining the most appropriate treatment for DEDs from the point of ocular surface inflammation.

It should be noted that this study did have several limitations. First, the components of the PPCS were not pathologically examined. Second, we did not confirm by rose Bengal staining whether the location of PPCS corresponded to that of decreased MUC16, as only fluorescein staining was used in this study. In our previous experience, we confirmed that the PPCS site can be stained by Rose Bengal. However, we have no information about whether the corneal epithelium behind the PPCS can be stained by rose Bengal. If this is the case, the possible attachment of mucin aggregates to the corneal epithelium with MUC16 being decreased can be somehow clarified. Third, this present retrospective study involved a relatively small number of subjects. Thus, further study may be needed to confirm the findings. Furthermore, the influence of topical treatment on the results of this present study needs to be considered from the aspect that the diagnosis of SS and the ocular surface examinations were conducted separately, and that the study may possibly have included cases diagnosed with SS and who might have previously had PPCS, however, it disappeared via the topical treatment at the time when the corneal fluorescein staining was performed. However, in regard to the relationship between PPCS manifestation and the results of the ocular surface examinations, there was no need to take the influence of topical treatment into consideration, as this present study examined the causal relationship between the manifestation of PPCS and the other ocular surface findings at a specific time-point.

## 5. Conclusions

In this study, the clinical features of two groups of DEDs presenting PPCS (PPCS group) and SPK without PPCS (non-PPCS group) were compared. Our findings revealed that the PPCS group showed significantly less reflex tear secretion, more severe CED, and a greater prevalence of SS than the non-PPCS group. These findings suggest that DEDs and dry eye patients with PPCS may not only indicate an association with SS, but also the mechanisms of dry eye associated with SS involving inflammation and delayed tear clearance.

## Figures and Tables

**Table 1:** Summary of the examinations of the ocular surface and the prevalence of Sjögren’s syndrome.

Examinations	PPCS Group (*n* = 35)	Non-PPCS Group (*n* = 30)	*p*-Value
TMR (mm)	0.18 ± 0.08	0.22 ± 0.09	0.12
SG (number of eyes) (Grade 1, 2, 3, 4, and 5)	16, 4, 6, 6, 3	20, 6, 0, 1, 3	0.07
NIBUT (seconds)	3.0 ± 2.2	4.4 ± 3.4	0.13
FBUT (seconds)	1.8 ± 1.3	2.9 ± 2.8	0.19
CED	5.3 ± 3.9	4.3 ± 4.5	0.12
CjED	3.1 ± 1.9	1.3 ± 1.6	0.0001
Schirmer 1 test (mm)	5.6 ± 7.4	14.7 ± 11.4	0.0005
Prevalence of SS (%)	21 (60)	5 (16.7)	0.0004

PPCS: patchy pattern corneal staining; TMR: tear meniscus radius; NIBUT: noninvasive breakup time; FBUT: fluorescein breakup time; CED: corneal epithelial damage; CjED: conjunctival epithelial damage; SS: Sjögren’s syndrome.

## Abbreviations

ATDDE	aqueous tear deficient dry eye
CED	corneal epithelial damage
CjED	conjunctival epithelial damage
CMP	corneal mucus plaque
DEDs	dry eye diseases
FBUT	fluorescein breakup time
MGD	meibomian gland dysfunction
NIBUT	noninvasive breakup time
PP	patchy pattern
PPCS	patchy pattern corneal staining
SG	spread grade
SPK	superficial punctate keratopathy
SS	Sjögren’s syndrome
ST1	Schirmer 1 test
TMR	tear meniscus radius
TFLL	tear film lipid layer
